# Key Factors Affecting Ambulatory Care Providers’ Electronic Exchange of Health Information With Affiliated and Unaffiliated Partners: Web-Based Survey Study

**DOI:** 10.2196/12000

**Published:** 2019-11-07

**Authors:** John C Pendergrass, Ranganathan Chandrasekaran

**Affiliations:** 1 Operations Management and Information Systems Northern Illinois University DeKalb, IL United States; 2 Center for Health Information Management and Systems University of Illinois at Chicago Chicago, IL United States

**Keywords:** health information exchange, ambulatory care information systems, ambulatory care facilities

## Abstract

**Background:**

Despite the potential benefits of electronic health information exchange (HIE) to improve the quality and efficiency of care, HIE use by ambulatory providers remains low. Ambulatory providers can greatly improve the quality of care by electronically exchanging health information with affiliated providers within their health care network as well as with unaffiliated, external providers.

**Objective:**

This study aimed to examine the extent of electronic HIE use by ambulatory clinics with affiliated providers within their health system and with external providers, as well as the key technological, organizational, and environmental factors affecting the extent of HIE use within and outside the health system.

**Methods:**

A Web-based survey of 320 ambulatory care providers was conducted in the state of Illinois. The study examined the extent of HIE usage by ambulatory providers with hospitals, clinics, and other facilities within and outside their health care system–encompassing seven kinds of health care data. Ten factors pertaining to technology (IT [information technology] Compatibility, External IT Support, Security & Privacy Safeguards), organization (Workflow Adaptability, Senior Leadership Support, Clinicians Health-IT Knowledge, Staff Health-IT Knowledge), and environment (Government Efforts & Incentives, Partner Readiness, Competitors and Peers) were assessed. A series of multivariate regressions were used to examine predictor effects.

**Results:**

The 6 regressions produced adjusted R-squared values ranging from 0.44 to 0.63. We found that ambulatory clinics exchanged more health information electronically with affiliated entities within their health system as compared with those outside their health system. Partner readiness emerged as the most significant predictor of HIE usage with all entities. Governmental initiatives for HIE, clinicians’ prior familiarity and knowledge of health IT systems, implementation of appropriate security, and privacy safeguards were also significant predictors. External information technology support and workflow adaptability emerged as key predictors for HIE use outside a clinic’s health system. Differences based on clinic size, ownership, and specialty were also observed.

**Conclusions:**

This study provides exploratory insights into HIE use by ambulatory providers within and outside their health care system and differential predictors that impact HIE use. HIE use can be further improved by encouraging large-scale interoperability efforts, improving external IT support, and redesigning adaptable workflows.

## Introduction

### Background

Health information exchange (HIE) is the electronic sharing of patient-level clinical health information among health care organizations, providers, and practice settings. HIE allows sharing of health information across organizational and geographic boundaries, making critical patient information available whenever and wherever needed [1,2]. HIE provides clinicians timely access to more complete patient information that is scattered across a fragmented US health care network. Ultimately, HIE can greatly improve quality of care, enhance patient safety, and reduce overall costs.

Evidence about the benefits of HIE is encouraging [3]. Studies on HIE outcomes have reported modest to moderate reductions in duplicate laboratory and radiology testing [4,5], better identification of medication discrepancies, and improved medication reconciliation [6]. HIE use has also decreased hospital readmissions and associated costs [7]. Faster access to patient information via HIE has enhanced emergency care and reduced costs [8]. From a business value viewpoint, effective use of HIEs has enhanced the resource utilization and productivity of providers, thereby improving their overall efficiency [9].

Despite such benefits, HIE usage has been low among ambulatory care providers [10]. In contrast to 76% hospitals who used HIE in 2014, only 42% of ambulatory care physicians engaged in any kind of HIE—within or outside their system. Furthermore, only 26% did so to share health information with external providers [11,12]. The relatively low HIE use among ambulatory clinics and the gap between HIE usage by hospitals and ambulatory care providers are concerning as the potential benefits of HIE will be hard to realize. Even among ambulatory clinics, there seem to be notable variations in the extent of electronic HIE within their health care system and with external providers. A health care system for an ambulatory clinic includes affiliated hospitals, providers, and other health entities who are connected through common ownership, or joint management, or some agreeable business arrangement [13].

HIE use in ambulatory care clinics can be complicated because each setting may have varying information needs, different levels of electronic health record (EHR) adoption, technological sophistication, and resource constraints. Although many technological, environmental, and organizational factors could affect the use of HIE [14], specific factors that affect HIE usage by ambulatory care providers remain underexplored. The influence of specific factors that cause variations in HIE usage within and outside the system is also not known. Exploring these factors is important to ensure the success of HIEs and to further promote their usage among ambulatory care providers. Our study addresses this gap by exploring the association between the key factors and HIE usage by ambulatory care providers. Our research objectives are 2-fold: (1) to assess the extent of HIE usage by ambulatory clinics to exchange health information with internal and external providers and (2) to examine the associations between key technology-organization-environment (TOE) factors and HIE usage within and outside the health care system.

### Key Factors Affecting Health Information Exchange Usage

**Figure 1:**
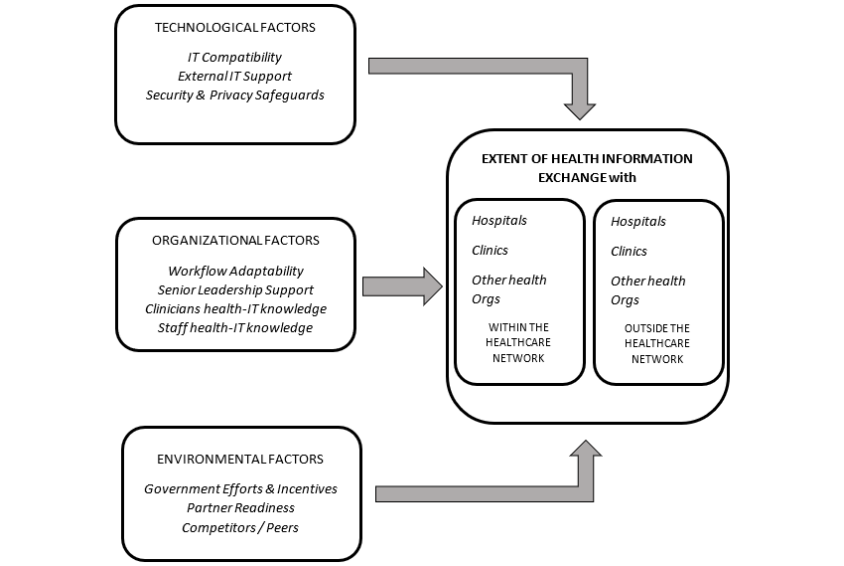
Key predictors of health information exchange use by ambulatory clinics: technology-organization-environment framework. IT: information technology; HIT: health information technology; Orgs: organizations.

Figure 1 presents a summary of key factors associated with ambulatory clinics’ HIE use—within or outside their system. The factors have been grouped based on the TOE framework [15] that has been widely used to understand the contextual factors associated with information technology (IT) usage in organizations.

The 2009 Health Information Technology for Economic and Clinical (HITECH) Act included a health care organization’s ability to engage in HIE as a criterion for Meaningful Use—the federal standard for incentives provided to clinicians and health care organizations for using EHR systems. Incentive payments for meaningful use in stage 2 require demonstrating actual HIE usage rather than merely possessing the ability to electronically exchange patient information. These efforts have resulted in a proliferation of HIE initiatives by hospitals as well as ambulatory care providers that range from minimally meeting the meaningful use requirements to more intense exchange of health care information [16,17]. Another key environmental factor influencing HIE use is the readiness level of partners. Ambulatory care clinics typically serve patients who also receive care from other partner hospitals, specialty clinics or laboratories and pharmacies, and these organizations could influence HIE use [18]. Ambulatory clinics can face interoperability challenges if partners differ in technological sophistication or use incompatible EHR systems. Competing clinics or providers can also influence HIE use [17,19]. Using HIE can help ambulatory clinics distinguish themselves from other competing clinics. However, in highly competitive markets, providers may be reluctant to share data because of fear of loss of patients to rival organizations [20]. Even among those sharing data, providers can restrict the type and amount of health care information that is shared [19].

Effective use of HIE is influenced by the ambulatory provider’s internal capabilities. The primary internal capabilities include workflow processes [16,21] and senior leadership support for HIE [22]. To enhance HIE usage, providers need to effectively incorporate HIE into current workflows and routines [23]. Workflows tend to differ across practice settings [21], and fusing them with HIE can be challenging [10]. Integrating HIE into current workflows can be achieved by (1) tweaking and redesigning existing workflows or (2) customizing the HIE-related information systems to fit in with the clinic-specific workflows. As the latter alternative can be costly, workflows in smaller physician practices will need considerable redesign for integration with HIE systems, and this can be a significant factor inhibiting HIE use in resource-constrained ambulatory settings [18]. Organizational leadership has been consistently found to be important to spearhead HIE initiatives among hospitals [24] and ambulatory clinics. HIE initiatives have been successful when senior leadership actively engages in HIE efforts and acts as organizational champions [25].

A provider’s IT infrastructure maturity and sophistication can greatly affect their HIE use. IT infrastructure includes the technological components as well as knowledge levels of key organizational stakeholders to effectively exploit IT to enhance business performance [26]. The fundamental building block for an interoperable, electronic HIE is a robust technological infrastructure and associated EHR system. In addition to flexible and compatible IT infrastructure, recognition of HIE’s potential and the knowledge levels of clinicians and administrative staff is an important factor associated with HIE usage [27]. An additional factor that can augment HIE usage is the external technical support provided to HIE users [27]. Ambulatory care providers have historically relied on external consultants and EHR vendors for training and technical support. With an evolving marketplace, identifying and gaining access to health care knowledgeable IT support has been challenging for several health care providers. Availability of quality and timely IT support can augment HIE use. An often-repeated concern in HIE relates to security and privacy of health information that is being exchanged. Implementing technological safeguards can greatly promote HIE use both within a health care system and with outside providers [17].

Building upon the TOE framework and prior studies, we conducted a survey study to assess the associations between these factors on HIE usage by ambulatory care providers within and outside their health care system.

## Methods

### Setting and Sample

This study draws from a population of ambulatory health care clinics from the state of Illinois. We partnered with 2 Illinois-based health information technology (HIT) regional extension centers (RECs) for recruiting ambulatory clinics to participate in our study. RECs are organizations that have received funding under the HITECH Act to assist health care providers with the selection and implementation of HIT. The primary constituency of RECs consists of small- and mid-sized practices that seek education and assistance in evaluating and implementing HIT. In addition, a list of Illinois rural health clinics and other ambulatory care providers was obtained from the Illinois Department of Public Health. The data were collected through a Web-based survey of office-based physicians in the practice. The Institutional Review Board of the University of Illinois at Chicago approved the study protocol. All participants provided informed consent. Respondents were provided a financial incentive to complete the Web survey.

### Survey Questionnaire

The survey questionnaire was constructed using items from several existing instruments that measured HIE usage and perceptions about the key TOE factors associated with HIE usage. We also used relevant items on the contextual factors from relevant HIT and management information systems literature. Multimedia Appendix 1 lists the survey items along with sources from literature. As most of the questionnaire items for survey were adapted from the prior literature, we gave careful consideration to the content validity of the measures. A total of 5 subject matter experts carefully assessed the survey items and wording of the items in the questionnaire. On the basis of their feedback, minor changes were made to the wording and design of the questionnaire. Then, the questionnaire was pretested with 6 clinicians from ambulatory clinics, and based on their feedback, minor changes were made to the survey.

In the survey, we specified HIE to mean any electronic form of data exchange—excluding fax machines—including any computer-to-computer interface (such as an EHR system) or a Web portal. To assess HIE usage, we asked respondents about their level of usage (coded as 0 for no usage, 1 for partial usage, and 2 for completely using electronic exchange) for sharing different types of health information (patient demographics, referrals, clinical orders, care summaries, physician notes or medication lists, radiology results, and laboratory results) within and outside their health care system. Respondents indicated HIE usage (0-2) for each health information type for 3 partner contexts—hospitals, clinics, and other health care organizations—both within and outside their system. For each ambulatory clinic, aggregate HIE usage scores were computed for each of the 3 partner contexts, that is, we computed 6 scores (ranging from 0 to 14) indicating HIE usage with hospitals, clinics, and others within their health care system, and similarly for the 3 partner contexts outside a clinic’s health care system.

Questions on the predictors (survey items are shown in Multimedia Appendix 1) captured the perceptions of respondents on the influence of technological factors (eg, compatibility of IT systems, access to external IT support, and security safeguards for exchanging health information), organizational factors (eg, vision of senior leadership on HIE, adaptability of workflows, and clinician and staff HIT knowledge), and environmental factors (eg, government regulations and incentives, competition, and readiness of partners) on HIE usage. Questions about perceptions used 5-point Likert scale responses ranging from *1, strongly disagree,* to *5, strongly agree*. Mean scores were computed for each of the predictors (Table 1). We also included questions on the ambulatory clinic’s demographic information such as the number of clinicians, practice specialty (family medicine, pediatrics, urgent care, obstetrics and gynecology, surgical, and other), type of setting (solo, group, primary care, and specialty care), and ownership status of the clinic (provider owned, hospital or system owned, or mixed).

**Table 1:** Descriptive statistics of technology-organization-environment predictors.

Predictor factors	Value, mean (SD)
IT^a^ compatibility	3.65 (0.89)
External IT support	3.37 (0.69)
Security safeguards	3.81 (0.88)
Senior leadership support	3.35 (0.76)
Workflow adaptability	3.27 (0.65)
Clinician HIT^b^ knowledge	3.77 (0.85)
Staff HIT knowledge	3.67 (0.74)
Government initiatives	3.35 (0.71)
Competitor and peer influence	3.01 (0.83)
Partner readiness	3.48 (0.94)

^a^IT: information technology.

^b^HIT: health information technology.

### Data Analysis

Demographic characteristics of respondent clinics were assessed using frequencies and percentages. For perceptive measures, wherever multiple items were defined a priori to reflect a factor, we took respondents’ average scores across respective items to create a summary score for each factor: influence of government, competition, and peers; HIT knowledge levels of clinicians and staff; workflow adaptability; security safeguards; and external IT support (see Table 1). All the statistical analyses were performed using IBM SPSS version 22 running on a Windows 10 platform.

For multiitem measures, we assessed the reliability (ie, internal consistency) and validity (ie, convergent and discriminant). Cronbach alpha is a standard way to perform a reliability analysis [28], and a range of .70 to .80 is considered acceptable. The Cronbach alpha values of our constructs ranged from .710 to .795 and hence were acceptable. Then, we performed a principal component analysis to assess validity. To assess convergent validity, the degree to which a measure is correlated with other measures that it is theoretically predicted to correlate with, we evaluated the loading of each item onto their specified factor. All loadings were above alpha=.70. We compared the coefficients of the indicators with the standard errors (where the loadings should be at least twice as much as the standard error) and assessed the *t* statistic, which were all significant at *P*=.05. To assess discriminant validity, we checked cross-factor correlations against the square root of the average variance extracted (AVE) of each factor. As correlations were all smaller than the square root of AVE, we concluded that discriminant validity was not a problem.

To explore the associations between the TOE factors and HIE usage, we performed a series of linear multivariate regressions. A series of 6 regressions were run with HIE usage as the dependent variable: one for each of the three partner contexts (hospitals, clinics, and others), both within and outside a health care system. Collinearity issues were also diagnosed by the examined variance inflation factor scores, which were all below permissible levels.

## Results

### Descriptive

Of the 2949 recruitment emails and faxes sent, 383 completed the survey for an overall response rate of 12.98% (383/2949). Moreover, 63 clinics indicated that they did not use any kind of HIE at all and were excluded from our analysis, reducing our usable sample size to 320.

Table 2 shows respondent demographics by number of clinical providers, clinic ownership, practice specialty, and type of setting. Over half (53.0%, 171/320) of the clinics have 10 or fewer providers, 43.0% (136/320) are wholly provider owned, over two-thirds (68.4%, 219/320) are either family medicine or pediatric clinics, and nearly half (48.1%, 154/320) constitute a primary clinic setting (either solo or group practice).

**Table 2:** Characteristics of respondent ambulatory clinics.

Clinic characteristics		Value, n (%)
**Number of providers**		
	1-2	41 (13)
	3-5	36 (11)
	6-10	94 (29)
	More than 10	149 (46.6)
**Practice setting**		
	Solo primary care	68 (21)
	Solo specialty care	104 (32.5)
	Group primary care	86 (27)
	Group specialty care	62 (19)
**Practice type**		
	Family medicine	101 (31.6)
	Pediatrics	118 (36.9)
	Urgent care	52 (16)
	Surgical	15 (5)
	Obstetrics/gynecology	25 (8)
	Others	9 (3)
**Clinic ownership**		
	Wholly physician/provider owned	136 (43.0)
	Wholly owned by hospital, health care system, HMO^a^, etc	123 (38.9)
	Partially owned by hospital, health care system, HMO, etc	47 (15)
	Others	10 (3)

^a^HMO: health maintenance organization.

Table 3 reports the nature of health information electronically shared by Illinois ambulatory clinics by data type for the three contexts both within and outside of the health care system. The mean and standard deviation of the independent variables is shown as well. Over half of the respondent clinics indicated sharing basic patient demographic information with providers within and outside of their system. However, only around one-third of ambulatory care providers exchanged care summary documents or referrals with other providers within and outside their system. About 40% of clinics indicated exchanging laboratory results and radiology reports with other providers. Electronic exchange of referrals ranged from 44.4% (142/320) to 52.5% (168/320) within the health care system and 35.9% (115/320) to 39.4% (136/320) outside the health care system.

**Table 3:** Number and percentage of providers exchanging information electronically by data type.

Measure	Within health care system			Outside health care system		
	Hospitals	Clinics	Others^a^	Hospitals	Clinics	Others^a^
Independent variable, mean (SD)	4.92^b^ (3.56)	5.17^b^ (3.67)	5.28^b^ (3.69)	4.35^b^ (3.06)	4.57^b^ (3.11)	4.68^b^ (2.95)
Patient demographics, n (%)	169 (52.8)	178 (55.6)	168 (52.5)	176 (55.0)	171 (53.4)	186 (58.1)
Referrals, n (%)	142 (44.4)	168 (52.5)	163 (50.9)	115 (35.9)	126 (39.4)	118 (36.9)
Clinical orders, n (%)	101 (31.6)	125 (39.1)	103 (32.2)	91 (28)	109 (34.1)	112 (35.0)
Clinical/summary care records, n (%)	107 (33.4)	124 (38.8)	121 (37.8)	98 (31)	118 (36.9)	120 (37.5)
Medication history and/or physician notes, n (%)	121 (37.8)	139 (43.4)	110 (34.4)	96 (30)	116 (32.3)	119 (37.2)
Laboratory results/reports, n (%)	125 (39.1)	117 (36.6)	142 (44.4)	124 (38.8)	119 (37.2)	130 (40.6)
Radiology results/reports, n (%)	124 (38.8)	124 (38.8)	133 (41.6)	137 (42.8)	128 (40.0)	130 (40.6)

^a^Others refer to laboratories, pharmacies, and other care facilities.

^b^Paired sample *t* tests confirmed significant differences in means between the 2 groups, *P*<.01.

### Predictors of Health Information Exchange Use

Table 4 presents the results of the 6 multivariate regression models, with panel 1 depicting the regression results for HIE use by Illinois clinics with hospitals, clinics, and other entities *within* their health care system and panel 2 reporting results for HIE use with hospitals, clinics, and others *outside* the Illinois clinics’ health care system. All the 6 models had statistically significantly adjusted R-squared values ranging from 0.44 to 0.63. Thus, our models were able to explain 43% to 63% of the HIE usage by sample clinics. Among the environmental factors, as presented in Table 4, partner readiness is the strongest predictor of HIE usage across all the 6 regression models. Government efforts for promoting HIE are also significantly associated with HIE with internal and external entities. According to panel 1, competitor or peer health organizations exert a negative influence on a clinic’s HIE use within the system.

Among the organizational factors examined, clinician’s knowledge of HIT systems emerged as a strong predictor across all 6 regression models, indicating that clinics with providers who used electronic medical records (EMRs) and other health technologies more are likely to see greater HIE usage. Moreover, senior leadership support for HIE is also a significant predictor of HIE use in 5 of the 6 models. Per panel 2, workflow adaptability is a significant predictor of HIE use for exchanging information with external clinics and other health entities. Panel 2 also indicates knowledge of ambulatory clinic’s staff to be negatively associated with HIE use with external clinics and external health entities. Taken together, providers’ knowledge and experience with HIT systems are more important to promote HIE usage rather than that of support staff in a clinic.

Among the technological factors, implementation of appropriate security safeguards emerged as a significant predictor of HIE use across all 6 regression models. Per panel 2, external IT support to ambulatory clinics is an important predictor of electronically exchanging health information with *external* hospitals, clinics, and other health entities. Compatibility of existing IT infrastructure is a significant predictor for exchanging health information with other entities within the clinics’ health care system.

Examining the clinic characteristics that were associated with HIE use, wholly provider-owned independent clinics, smaller clinics with 1 or 2 providers, family medicine practices, and solo primary care clinics seem to engage in more HIE use within as well as outside the health care system.

**Table 4:** Regression coefficients: key predictors of health information exchange use by ambulatory clinics.

Predictor variables			HIE^a^ within health care system			HIE outside health care system			
			Hospitals	Clinics	Others		Hospitals	Clinics	Others
**Environmental factors**									
	Government initiatives for HIE		0.17^b^	0.12^c^	0.10^d^		0.24^b^	0.27^b^	0.17^b^
	Partner readiness		0.36^b^	0.24^b^	0.31^b^		0.24^b^	0.27^b^	0.21^b^
	Peers and competitors		−0.24^b^	−21^b^	−0.15^c^		—^d^	—	—
**Organizational factors**									
	Senior leadership support for HIE		0.13^e^	0.16^c^	—		0.16^c^	0.12^e^	0.21^b^
	Workflow adaptability		—	—	—		—	0.09^e^	0.10^e^
	Clinicians HIT^f^ knowledge		0.14^c^	0.17^b^	0.14^c^		0.10^e^	0.10^c^	0.14^b^
	Staff HIT knowledge		—	—	—		—	−0.11^c^	−0.12^c^
**Technological factors**									
	IT^g^ compatibility		—	—	0.14^c^		—	—	—
	External IT support		—	—	—		0.13^c^	0.14^b^	0.09^e^
	Security safeguards		0.13^c^	0.10^e^	0.11^e^		0.15^c^	0.17^b^	0.10^c^
**Clinic characteristics**									
	**Providers per clinic**								
		01-Feb	Reference	Reference	Reference		Reference	Reference	Reference
		03-May	—	—	—		−0.11^e^	−0.10^e^	—
		06-Oct	—	—	—		—	−0.15^c^	—
		More than 10	—	—	—		—	−0.15^c^	—
**Ownership**									
	Wholly or partly owned by hospital, HMO^h^, or health system		Reference	Reference	Reference		Reference	Reference	Reference
	Provider owned		—	0.10^e^	0.11^c^		—	—	0.36^b^
**Specialty**									
	Family medicine		Reference	Reference	Reference		Reference	Reference	Reference
	Pediatric care		−0.17^b^	−0.24^b^	−27^b^		−0.16^b^	−22^b^	−27^b^
	Urgent care		−0.12^c^	−0.15^b^	−0.12^c^		−0.11^c^	−0.20^b^	−0.19^b^
	Obstetrics/gynecology or surgical/others		−0.11^c^	−0.11^c^	−0.13^c^		−0.14^c^	−0.13^b^	−0.22^b^
**Setting**									
	Solo primary care		Reference	Reference	Reference		Reference	Reference	Reference
	Solo specialty care		−0.15^c^	−0.14^c^	−25^b^		−0.15^c^	−0.11^e^	−0.12^c^
	Group primary care		—	−0.12^e^	−0.20^c^		−0.18^b^	−0.13^c^	−22^b^
	Group specialty care		—	—	—		−0.13^e^	−0.11^c^	−0.12^c^
	Adjusted R-squared		0.47	0.45	0.44		0.50	0.63	0.61
	*F* test		12.46^b^	11.57^b^	10.84^b^		13.79^b^	22.79^b^	20.88^b^

^a^HIE: health information exchange.

^b^*P*≤.01.

^c^*P*≤.05.

^d^Not applicable.

^e^*P*≤.10.

^f^HIT: health information technology.

^g^IT: information technology.

^h^HMO: health maintenance organization.

## Discussion

### Analysis

To our knowledge, this is the first empirical study to examine predictors of HIE within and outside a health care system in ambulatory settings. Using data from 383 clinics, we examined TOE factors associated with the use of electronic HIE by small- and mid-sized clinics across the state of Illinois. Usage was examined for exchange partners within the clinic’s health care system, if any, and outside their health care system. For each system context, 3 categories of exchange partners were analyzed: hospitals, clinics, and other health care–related entities. Regression results varied by usage context, thereby revealing a differentiated understanding of the phenomena not previously examined.

Partner readiness emerged as the most significant predictor of HIE usage with all affiliates within as well as outside health care system. Governmental initiatives for HIE, clinicians’ prior familiarity and knowledge of HIT systems, and implementation of appropriate security and privacy safeguards are also significant predictors of all the 6 contexts examined.

A key factor influencing decisions about the use of a health technology is the knowledge about the capabilities, limitations, and consequences of using that technology. For clinicians, knowledge about HIT systems improves with prior and continued use of technologies. A significant relationship between clinician HIT knowledge and HIE usage was indicated for all contexts both within and outside the health care system. When exchanging patient information in traditional ways (eg, fax), the workflow often utilizes administrative staff for performing these functions. However, the use of electronic HIE puts capabilities and access to information instantly at the hands of clinicians. If clinicians are required to utilize EMR systems, and different forms of electronic HIE are tied to the EMR, then usage is dependent on the clinician’s knowledge of the EMR technology. In contrast, we found staff knowledge of HIT to be an insignificant predictor of electronic HIE usage within the health care system, but it was negatively related to HIE usage in 2 contexts outside the health care system. Staff knowledge might not be sufficient to promote electronic HIE, rather it is those clinicians who are adept at using EMR and other HIT systems who are likely to promote electronic HIE in ambulatory settings. Our survey assessed clinician perceptions regarding the knowledge of staff about HIT systems in general, and our findings show that generic knowledge of HIT systems might prove to be a negative factor for exchange of health information with unaffiliated entities. Our findings point to the need for additional HIE-specific training that may be required for staff in ambulatory clinics.

Peer practices, which include competition, indicated a negative and significant relationship for the 3 contexts within the clinic’s health care system but no statistical significance outside the health care system. Our findings regarding peer influence inhibiting HIE usage within a health care system underscore competitive concerns for provider organizations’ participation in HIE.

Workflow adaptability and external IT support exhibited significant positive association with HIT usage outside the clinic’s health care system. HIE within a clinic’s health care system might be relatively easier as the entities may not have much variability in terms of workflows and EMR systems. However, considerable variations in terms of HIT systems, processes, and workflows could impede electronic HIE with external partners. Managers and policy makers need to work toward more standardization of workflows and offer greater technical support to spur more HIE usage of clinics with partners outside their health care system.

Our findings confirm significant variations in HIE usage within and outside a clinic’s health care system. This finding coupled with the need for external IT support for HIE use with external providers underscores a broader interoperability problem in exchanging health information. For instance, the leading EMR vendor Epic Systems has been criticized for limiting the data exchange capabilities and for charging providers additional fees to enable data exchange with providers who use non-Epic systems [29]. In response to such criticisms and subsequent congressional hearings [30], several new interoperability improvement efforts have been proposed. These initiatives, though well intended, could further increase the fragmentation. Deliberate *information blocking* practices by EHR vendors and providers where they knowingly interfere with electronic exchange of health information for competitive purposes can also impede HIE use [31]. Ambulatory clinics could be coerced into implementing systems and document exchange practices by larger providers or vendors [32]. Recent federal efforts aimed at implementing and improving interoperability standards are moving in the right direction to promote HIE use by clinical providers, for instance, the support by the Office of the National Coordinator for Health Information Technology for the development of solutions using the Fast Health care Interoperability Resources standard [33].

### Limitations

A major limitation of this study stems from the sample being drawn from a single state. However, HIE efforts in the state of Illinois are not unlike those in many other states and, therefore, results may be typical. State-level efforts to improve HIE have varied across the country, and these could have differential impacts on HIE use by ambulatory providers. Another limitation pertains to self-reported data. We relied on perceptions of our respondents to capture our variables. We were unable to obtain specific objective measures of HIE use. More objective measures of HIE such as the number of records exchanged or volume of data exchanged could be a fruitful extension of our research.

### Conclusions

This study provides exploratory insights into HIE use by ambulatory providers within and outside their health care system and differential predictors that impact HIE use. By identifying key predictors, we have highlighted the importance of federal efforts, status of partners, and HIT knowledge of clinicians. HIE use by ambulatory providers can be further improved by encouraging large-scale interoperability efforts across the industry, improving external IT support, and redesigning adaptable workflows.

## Appendix

Multimedia Appendix 1 Questionnaire items and sources.

## Abbreviations

AVE: average variance extracted
EHR: electronic health record
EMR: electronic medical record
HIE: health information exchange
HIT: health information technology
HITECH: Health Information Technology for Economic and Clinical
IT: information technology
REC: regional extension center
TOE: technology-organization-environment
