# Supplementary material for: Key Factors Affecting Ambulatory Care Providers’ Electronic Exchange of Health Information With Affiliated and Unaffiliated Partners: Web-Based Survey Study
Source: JMIR Med Inform. 2019 Nov 7;7(4):e12000. doi: 10.2196/12000 (PMC6913753; doi:10.2196/12000)
Supplement: Multimedia Appendix 1 [file medinform_v7i4e12000_app1.pdf]

Appendix A: Questionnaire Items and Sources

Questions	Sources
**HIE Use**<br>*To what extent does your clinic engage in electronic change of the following health information with other hospitals within your healthcare system?*<br>*(0 = No electronic exchange; 1 = partial electronic exchange; 2 = entirely electronic exchange)*<br>● *Patient demographics*<br>● *Referrals*<br>● *Clinical orders*<br>● *Clinical/Summary care records (any format)*<br>● *Medication history and/or physician notes*<br>● *Lab results*<br>● *Radiology results*<br>[This question was repeated for clinics, and other health facilities, for both *inside* and *outside* a healthcare system]	[13,33] [34] [35]
**IT Compatibility**<br>(*1=Strongly Disagree, 5=Strongly Agree)*<br>● *Our clinic's information technology infrastructure is compatible with HIE.*	[36][26]
**External IT support**<br>(*1=Strongly Disagree, 5=Strongly Agree)*<br>● *IT support is available when needed.*	[35]
**Security Safeguards**<br>(*1= Strongly Disagree, 5=Strongly Agree)*	[35]

● *Our clinic is attentive to patient information security when exchanging with external parties.*	
**Senior Leadership Support**<br>(*1=Strongly Disagree, 5=Strongly Agree)*<br>● *Our clinical leadership considers the use of HIT (like EHR) important.*<br>● *Our clinical leadership has a well-defined vision of how Health IT will advance the goals our clinic.*<br>● *Our clinical leadership has a favorable view toward HIE*	[37][38]
**Workflow Adaptability**<br>(*1=Not adaptable at all, 5=Extremely adaptable)*<br>● *How adaptable is your clinical workflow to using electronic exchange methods?*	[35]
**Clinician HIT knowledge**<br>(*1=Strongly Disagree, 5=Strongly Agree)*<br>● *Our clinicians are quite knowledgeable about the health IT systems that we have.*	[39]
**Staff HIT knowledge**<br>(*1=Strongly Disagree, 5=Strongly Agree)*<br>● *Our staff (non-clinicians) are quite knowledgeable about health IT systems that we have.*	[39]
**Government Initiatives**<br>*(1=no influence; 5=very strong influence)*<br>*Extent to which the following factors have influenced your clinic's HIE use*<br>● *government regulations*<br>● *government incentives*<br>● *government agencies' (State-agencies, regional organizations and extension centers) efforts.*	[40]

**Competitor/Peer influence**<br>*(1=no influence; 5=very strong influence)*<br><br>*Extent to which the following factors have influenced your clinic's HIE use*<br><br>● *Peer clinics operating in your network.*<br>● *Competing clinics in your local area.*	[37] [38]
**Partner Readiness**<br>*(1=Strongly disagree; 5=strongly agree)*<br><br>● *Most of the specialists and other clinics we work with prefer electronic exchange of health information.*<br>● *Most of the hospitals we work with prefer electronic exchange of health information.*<br>● *Many health facilities we work with (e.g. labs, pharmacies, other centers etc.) prefer electronic exchange of health information.*	[37] [38]
